# Love of life and its association with well‐being in Iranian psychiatric outpatients

**DOI:** 10.1002/nop2.575

**Published:** 2020-08-07

**Authors:** Mahboubeh Dadfar, Ahmed M. Abdel‐Khalek, David Lester

**Affiliations:** ^1^ School of Behavioral Sciences and Mental Health‐Tehran Institute of Psychiatry International Campus School of Public Health, Student Committee of Education and Development Center (EDC) Iran University of Medical Sciences Tehran Iran; ^2^ Department of Psychology Alexandria University Alexandria Egypt; ^3^ Stockton University Galloway NJ USA

**Keywords:** Iran, love of life, psychiatric outpatients, psychological well‐being, spiritual well‐being

## Abstract

**Aim:**

The aims of this study were as follows: (a) to investigate the psychometric properties of the Love of Life Scale (LLS); (b) to explore sex differences in LLS scores; (c) to explore LLS correlations with spiritual health and psychological well‐being; and (d) to compare the mean LLS score with previous studies.

**Design:**

A cross‐sectional study.

**Methods:**

A sample of 191 Iranian psychiatric outpatients was recruited from clinics at the School of Behavioral Sciences and Mental Health (Tehran Institute of Psychiatry), which is affiliated with the Iran University of Medical Sciences. They responded to the Farsi versions of the LLS, the Spiritual Well‐Being Scale (SWS) and the World Health Organization‐five Well‐Being Index (WHO‐5).

**Results:**

Cronbach alpha for the LLS was 0.95, and a principal component analysis (PCA) of the LLS items extracted one component labelled "Love of life." The sex difference in LLS scores was not significant. All the correlations between the scales were statistically significant and positive. Patients who had high scores for their love of life had better spiritual health and psychological well‐being.

**Discussion:**

The present sample obtained a lower mean LLS score than college students from eight countries except one. Further research should explore predictors of the love of life.

## 1 INTRODUCTION

Liking and loving are an important topic in social psychology (Rubin, 1970, 1973; Rubin & McNeil, 1983), and love plays a key role in the communication between individuals (Baxter & Akkoor, 2008). More generally, Abdel‐Khalek (2007, 2013a) has argued that love of life is a sub‐component of the construct of subjective well‐being and it is relevant to the disciplines of positive psychology and health psychology. Love of life is defined as a positive evaluation of one's own life and involves holding on strongly to life, grasping at life, having a pleasurable attachment to life and having an appreciation for life. However, the love of life concept is not related to the Freudian concept of Eros or the life instinct (see Campbell, 1996).

Research has shown that love of life is positively associated with extraversion and negatively associated with psychoticism and neuroticism (Abdel‐Khalek, 2013a). Love of life is associated with having meaning in life, optimism, social support, mental and physical health, happiness, satisfaction with life, religiosity, subjective well‐being and hope (Abdel‐Khalek, 2007; Al‐ Ayoob, 2010; Dadfar, Lester, Turan, Beshai, & Unterrainer, 2019, 2020; Dadfar, Moghaddasi, Mohebi, Mohaghegh, & Eslami, 2018; El‐Nayal, 2009b; Simmons & Lehmann, 2013). Love of life is negatively associated with the wish to be dead (Dadfar, Lester, Atef Vahid, & Abdel‐Khalek, 2017b) and death anxiety (Dadfar, Abdel‐Khalek, Lester, & Atef Vahid, 2017a).

The Love of Life Scale (LLS), developed by Abdel‐Khalek in Arabic and English (2007), is a measure of this construct (Abdel‐Khalek, 2013b; Abdel‐Khalek & Lester, 2011; Simmons & Lehmann, 2013). It has also a Persian version (Atef Vahid, Dadfar, Abdel‐Khalek, & Lester, 2016).

Most previous studies on the LLS have been carried out using college students. Therefore, there is a need to explore the performance of the LLS with clinical cases. To the best of our knowledge, the LLS has not been administered to psychiatric outpatients. Furthermore, the studies on the LLS using the Farsi version with Iranian participants are rare.

There are specific applications of the present study for nursing practice. Based on the statistically significant negative associations between the LLS and psychopathology, mainly anxiety, depression and suicidal ideation, the score on the LLS could be used as one of the indicators to improvement after psychological interventions for patients, particularly in psychiatric nursing, psycho‐rehabilitation and mental health nursing, among other nursing branches. Therefore, the LLS could be useful in nursing education.

## 2 BACKGROUND

At a theoretical level, it is important to investigate whether the LLS behaves differently in psychiatric patients compared with university students. Because of the presence of symptoms of anxiety and depression, we hypothesize that the LLS scores of psychiatric patients would be lower than that of non‐clinical persons. Individuals with anxiety disorders may suffer from specific phobias, social anxiety, panic disorder, agoraphobia and general anxiety. Individuals with specific phobias are fearful or anxious about or avoidant of circumscribed objects or situations. Individuals with general anxiety disorder have persistent and excessive anxiety and worry that the individual finds difficult to control about various domains, including work and school performance. In addition, they experience physical symptoms, including restlessness or feeling keyed up or on edge; being easily fatigued; difficulty concentrating or their mind going blank; irritability; muscle tension; and sleep disturbance (American Psychiatric Association, 2013, pp. 189–190). Because of these symptoms and complaints, the sense of subjective well‐being, as well as love of life, may be negatively affected in individuals with anxiety disorders (Abdel‐Khalek, 2013a, 2018).

Individuals with depression suffer from some of the following symptoms: depressed mood, loss of interest or pleasure, hopelessness, changes in body weight, decrease or increase in appetite, insomnia or hypersomnia, psychomotor agitation or retardation, fatigue, feelings of worthlessness, diminished ability to think and thoughts of death (American Psychiatric Association, 2013, pp. 160–161). Symptoms and complaints like these may have a negative impact on the love of life.

The aims of the present study were (a) to investigate the psychometric properties of the Farsi version of the Love of Life Scale (LLS) with psychiatric outpatients, (b) to explore sex differences in LLS scores, (c) to explore LLS correlations with spiritual and psychological well‐being and (d) to compare the mean LLS scores of the present sample of psychiatric patients with the available results for the LLS in previous research by others.

## 3 METHODS

### 3.1 Participants

A convenience sample of 191 Iranian psychiatric outpatients was recruited from clinics at the School of Behavioral Sciences and Mental Health (Tehran Institute of Psychiatry), which is affiliated with the Iran University of Medical Sciences. The sample size was calculated using Cochran's formula. To estimate the sample size, three issues need to be studied (the level of precisions, confidence or risk level and the variability). The less variable (more homogeneous) a population, the smaller the sample size. For reason, the patients’ population we were studying was small, we modified the sample size by using this equation: *n* = [n0/(1+((n0–1)/N)). The patients were invited to voluntarily participate in the study after they completed a psychiatric interview with one psychiatrist. The objective of study was explained to the patients. The scales contained a cover letter, explaining all the ethical considerations (confidentiality, anonymity, informed written consent and the right to withdraw) as well as an explanation of the research procedure. The response rate was 100%.

### 3.2 Measures

#### 3.2.1 The Love of Life Scale (LLS)

The LLS, developed by Abdel‐Khalek (2007), is a 16‐item self‐report scale. Each item is answered on a five‐point Likert‐type scale: No (1); A little (2); Moderate (3); Much (4); and Very much (5). All the items are keyed positively. The total score can range from 16 (strong disagreement with all items)–80 (strong agreement with all items). High scores indicate a high love of life, while low scores indicate a low love of life. The LLS was written originally in Arabic and has an equivalent English and Farsi versions. It has been administered to Egyptian, Kuwaiti, Lebanese, Qatari, Iranian, Algerian, Indian, Malaysian and American university students, as well as to adolescents, adults and older people and to cancer and MS patients (Abdel‐Khalek, 2007, 2013a; Abdel‐Khalek & Zine El‐Abiddine, 2019; Al‐Ayoob, 2010; Atef Vahid et al., 2016; Dadfar, Abdel‐Khalek, et al., 2017a; Dadfar, Lester, et al., 2017b; El‐Nayal, 2009a, 2009b). In Abdel‐Khalek's study (2007), the LLS had high internal consistency (Cronbach *α* = 0.91) and test–retest reliability (*r* = .81). A principal components analysis extracted three factors labelled: positive attitude towards life, happy consequences of love of life and meaningfulness of life, with moderate inter‐factor correlations.

#### 3.2.2 The Spiritual Well‐Being Scale (SWS)

The SWS, developed by Paloutzian and Ellison (1991), contains 20 items. Each item is answered on a six‐point Likert‐type scale. Ten items measure religious well‐being (a religious element and a sign of relationship with a superior authority, e.g. God), and 10 items measure existential well‐being (a psychosocial element and a sign of a person's feelings about who he/she is, what and why he/she does and where he/she belongs). The scores of religious and existential well‐being subscales ranged 10–60, and total scores ranged 20–120. The SWS has a Persian version (Biglari Abhari, Fisher, Kheiltash, & Nojomi, 2018; Kazemzadeh Atoofi, Dadfar, Turan, & Behnam, 2019) and a Portuguese version (de Araujo Toloi et al., 2016). Paloutzian and Ellison (1991) reported test–retest reliability coefficients for the religious well‐being, existential well‐being subscales and the total SWS were 0.63, 0.86, 0.93 and Cronbach α were 0.91, 0.93 and 0.91, respectively. Biglari Abhari et al. (2018) found test–retest reliability for the SWS was 0.98 and Cronbach α was 0.85 in an Iranian sample.

#### 3.2.3 The World Health Organization‐five Well‐Being Index (WHO‐5)

The WHO‐5 has a Persian version (Dadfar, Momeni Safarabad, Asgharnejad Farid, Nemati Shirzy, & Ghazie pour Abarghouie, 2018). The WHO‐5 is a five‐item instrument used to screen for depression and is useful in a clinical context. The items are rated on a six‐point Likert scale. Dadfar, Momeni Safarabad, et al. (2018) found Cronbach's *α* for the WHO‐5 was 0.91 among Iranian psychiatric outpatients.

Previous studies have reported high reliability and validity of the Farsi versions of the three last mentioned scales.

### 3.3 Procedure

Participants responded to the Farsi versions of the LLS, the SWS and the WHO‐5 in individual sessions. Data were collected from 31 January 2018–21 July 2018.

### 3.4 Data analysis

For determination of the normality of the data and equality of variances, the Kolmogorov–Smirnov test and Levene's test were used, respectively. The data were analysed with descriptive statistics (mean, standard deviations), *t* tests, Pearson correlation coefficients and a principal components factor analysis to identify the number of factors to be retained. The criterion of eigenvalues greater than or equal to 1.0 was followed, and the Varimax orthogonal rotation of axes was adopted. The SPSS/WIN (SPSS & Inc., 2009) 23. 0 program was used.

## 4 FINDINGS

Table 1 reports demographic and clinical characteristics of patients.

**Table 1:** Demographic and clinical characteristics of participants

Variables	*M* (*SD*)
Age	31.90 (10.67)
Duration of disorder	7.13 (7.06)

	*N* (%)
Sex	
Male	35 (18)
Female	156 (82)
Marital status	
Single	100 (53)
Married	91 (47)
Education level	
Bachelor's degree	69 (36)
Other	122 (64)
Diagnosis	
Anxiety	79 (41.4)
Depression	72 (38)
Mixed anxiety depression	29 (15.1)
Missing	11 (5.5)

Table 2 reports the item‐total correlations for the LLS. These correlations ranged from 0.653–0.829 (significant at 0.01 level). Cronbach's alpha was 0.95. The mean total score for the LLS was 51.02 (*SD* = 15.09). For the factor analysis, the KMO was 0.945 and the Bartlett's test chi‐square was 2,227.315 (*df* = 120, *p* > .000). A principal component analysis (PCA) extracted only one factor, accounting for (58.68% of the total variance). It may be labelled Love of Life.

**Table 2:** Reports the item‐total correlations, factor loadings (>0.50), and Cronbach's α for the LLS

LLS items	Pearson *r* with LLS total score	Factor 1
1. Life is full of pleasures.	.749*	0.754
2. There are many things that make me love life.	.776*	0.780
3. Love of life adds to its beauty.	.653*	0.651
4. Life deserves to be loved.	.768*	0.771
5. Love of life makes me happy.	.783*	0.782
6. Life seems beautiful and wonderful to me.	.812*	0.814
7. I look at life from its beautiful side.	.739*	0.744
8. Love of life gives me hope.	.785*	0.783
9. I would like to have a long life to achieve what I hope for.	.712*	0.701
10. Love of life brings me satisfaction.	.785*	0.782
11. Life is a treasure we should guard.	.778*	0.777
12. Life is beautifully meaningful.	.785*	0.788
13. Life is a blessing whose value we should appreciate.	.777*	0.776
14. I realize that my existence in this life has great meaning.	.829*	0.828
15. I always have a wonderful feeling of loving life.	.817*	0.821
16. I like to be optimistic about life.	.685*	0.681
Eigenvalue		9.39
% of total variance		58.68
Correlations with Other Scales		
Spiritual well‐being scale (SWBS)	.67*	
World health organization‐five well‐being index (WHO‐5)	.53*	
Mean score (*SD*) for the LLS	51.02 (15.09)	
Cronbach's *α* for the LLS	.95	

* Two‐tailed *p* < .001.

Men and women did not differ in LLS scores (Mean = 48.50, *SD* = 14.43 for men; and Mean = 51.64, *SD* = 15.24 for women, *t* = 1.09, n.s). There were positive correlations between LLS scores and spiritual well‐being (*r* = .67) and psychological well‐being (*r* = .53) scores.

Table 3 reports the mean LLS scores for the present sample of psychiatric patients and for college students in previous studies. Inspection of this table indicates that the mean LLS score of the present sample is lower than that of college students in all countries except for Egyptian females.

**Table 3:** Descriptive statistics for the Love of Life Scale in the present study and previous results on college students

Samples	Men		Women	
	*M*	*SD*	*M*	*SD*
Present study	48.5	14.4	51.6	15.2
Previous results				
Iran	–	–	61.1	11.4
Algeria	55.9	13.3	57.6	12.1
Egypt	51.5	10.8	50.7	11.5
Kuwait	58.1	11.8	55.6	11.4
Lebanon	54.2	13.2	55.2	12.3
Palestinian	53.0	12.7	54.3	14.6
Qatar	59.0	10.4	58.5	11.4
India	58.6	12.6	61.6	7.8
Malaysia	64.6	9.9	64.4	8.9
USA	63.2	10.5	(Men & women)	

## 5 DISCUSSION

The present study indicated that the Farsi version of the LLS is a reliable scale. The Cronbach alpha reliability of the LLS was 0.95, and the item‐total correlations were moderate to high. These findings were consistent with previous studies of the reliability of the LLS in samples from Algeria, India and Palestine (Abdel‐Khalek & Singh, 2019; Abdel‐Khalek & Zine El‐Abiddine, 2019; Al‐Arja, 2018). For example, Abdel‐Khalek (2007) reported high internal consistency (0.91) and test–retest reliability (0.81) for the scale with an Egyptian sample. Atef Vahid et al. (2016) found a Cronbach *α* of 0.94 and the test–retest reliability of 0.85 after a 1‐week interval in a sample of Shia Muslim Iranian female university students.

The PCA extracted one component labelled "Love of life." Abdel‐Khalek and Zine El‐Abiddine (2019) extracted a single factor labelled “Love of life and optimism versus pessimism,” and a study by Abdel‐Khalek and Singh (2019) in Indian Hindu and Muslim college students extracted a single factor labelled “Well‐being and religiosity” accounting for 60.37% of the total variance. However, Atef Vahid et al. (2016) extracted two factors for the LLS: (a) positive attitude towards life and happy consequences of love of life and (b) meaningfulness of life. Thus, in general, research supports the existence of a homogenous LLS.

In a sample of Palestinian students, Al‐Arja (2018) found that love of life was not correlated with gender or their parents’ academic level. She found also that the love of life mean score was significantly higher in Christian than in Muslim students. Students who were resident in villages had higher love of life mean score compared with those living in refugee camps. Perhaps as a result of the politically and economically unstable and harsh situations for the Palestinian students, their mean LLS score was lower than the scores of Kuwaiti and Lebanese students.

Associations between love of life and both spiritual well‐being and psychological well‐being were significant and positive for the psychiatric patients in the present study. Patients who had more love of life had better spiritual health and psychological well‐being. This finding is consistent with results from other studies. Abdel‐Khalek (2013c, 2015) found that religious college students from Egypt and Qatar reported higher subjective well‐being. In a similar vein, Atef Vahid et al. (2016) reported positive correlations between scores on the LLS and the Oxford Happiness Questionnaire, the Satisfaction with Life Scale, the General Self‐Efficacy Scale and the Adult Hope Scale. In addition, negative correlations were reported with scores on the Wish to be Dead Scale and the Kessler Psychological Distress Scale. Abdel‐Khalek (2007) reported positive correlations of LLS scores with happiness, optimism, self‐esteem, hope, satisfaction with life and extraversion. Using the LLS and the Multidimensional Inventory for Religious Spiritual Well‐Being (MI RSWB 48), Bahrami et al. (2016) found a significant difference between love of life and forgiveness scores before and after participation in the religious spiritual retreat of I’tikaf among Shia Muslim Iranian female university students.

Dadfar, Abdel‐Khalek, et al. (2017a) reported a non‐significant negative correlation between scores on the LLS and on the Arabic Scale of Death Anxiety (ASDA) in Muslim Iranian college students. Dadfar, Momeni Safarabad, et al. (2018) found a significant positive association between scores on the LLS and the Self‐Rating Scale of Happiness in Shia Muslim Iranian patients with multiple sclerosis (MS). Abdel‐Khalek and Zine El‐Abiddine (2019) found that LLS scores were significantly associated with scores on the Arabic Scale of Happiness, the Satisfaction with Life Scale, the Arabic Scale of Optimism (positive) and pessimism (negative). Abdel‐Khalek (2018) found that LLS scores were negatively associated with suicide ideation, hopelessness and neuroticism, indicating convergent and divergent validity of the LLS. The present results, therefore, are consistent with other construct validity studies of the LLS.

The present study showed that the sex difference in LLS scores was not significant in this clinical sample. This finding is consistent with other studies in non‐clinical samples of college students from Kuwait, Qatar, Egypt, Lebanon, Algeria and India, as well as in Kuwaiti Muslim adolescents and middle‐aged adults (e.g. Abdel‐Khalek, 2007, 2012, 2013a, 2015; Abdel‐Khalek & Zine El‐Abiddine, 2019).

It was expected that the present sample of psychiatric outpatients would obtain a lower mean score on LLS in comparison with college students from eight countries and this was confirmed, except for the Egyptian participants who are possibly facing economic hardships.

The present study had some limitations. Its findings were based only on Iranian, Muslim, psychiatric outpatients. The psychiatric diagnosis of the patients was not taken into account, and there may be differences in the results between, for example, patients with schizophrenia and those with affective disorders. Further research should use a representative sample derived from the general population in Iran to test the replicability of the one‐factor solution for the LLS. Further research might also explore predictors of love of life.

## 6 CONCLUSION

The current investigation has successfully achieved its objectives; that is, the LLS had good internal consistency. A factor analysis identified one factor which is consistent with some previous studies and indicates a good degree of homogeneity of the item content. Consistent with previous findings, the sex differences were not statistically significant. The associations of LLS with spiritual well‐being and general well‐being were statistically significant and positive. As predicted, the mean score on the LLS among the present sample of psychiatric patients was lower than most previous studies carried out on non‐clinical samples from several countries. All in all, the love of life concept may be considered one of the major constructs related to subjective well‐being. Furthermore, we suggest developing a programme to promote love of life among patients. This programme would ameliorate physical and psychological symptoms and would improve their social adjustment. This proposed programme may be incorporated into psychotherapeutic procedures with patients.

## CONFLICT OF INTEREST

The authors declare that there is no funding for the study and they have no conflict of interest regarding the publication of this paper.

## AUTHOR CONTRIBUTIONS

All authors have agreed on the final version and meet at least one of the following criteria [recommended by the ICMJE (http://www.icmje.org/recommendations/)]: substantial contributions to conception and design, acquisition of data or analysis and interpretation of data; and drafting the article or revising it critically for important intellectual content.

## ETHICAL APPROVAL

The Institutional Review Board Research of Iran University of Medical Sciences approved this study.
